# CCBE1 promotes GIST development through enhancing angiogenesis and mediating resistance to imatinib

**DOI:** 10.1038/srep31071

**Published:** 2016-08-10

**Authors:** Guang-Ang Tian, Chun-Chao Zhu, Xiao-Xin Zhang, Lei Zhu, Xiao-Mei Yang, Shu-Heng Jiang, Rong-Kun Li, Lin Tu, Yang Wang, Chun Zhuang, Ping He, Qing Li, Xiao-Yan Cao, Hui Cao, Zhi-Gang Zhang

**Affiliations:** 1 State Key Laboratory of Oncogenes and Related Genes, Shanghai Cancer Institute, Ren Ji Hospital, School of Medicine, Shanghai Jiao Tong University, Shanghai, 200240, P.R. China; 2 Shanghai Medical College of Fudan University, Shanghai, 200032, P.R. China; 3 Department of General Surgery, Ren Ji Hospital, School of Medicine, Shanghai Jiao Tong University, Shanghai, 200127, P.R. China

## Abstract

Gastrointestinal stromal tumor (GIST) is the most major mesenchymal neoplasm of the digestive tract. Up to now, imatinib mesylate has been used as a standard first-line treatment for irresectable and metastasized GIST patients or adjuvant treatment for advanced GIST patients who received surgical resection. However, secondary resistance to imatinib usually happens, resulting in a major obstacle in GIST successful therapy. In this study, we first found that collagen and calcium binding EGF domains 1 (CCBE1) expression gradually elevated along with the risk degree of NIH classification, and poor prognosis emerged in the CCBE1-positive patients. *In vitro* experiments showed that recombinant CCBE1 protein can enhance angiogenesis and neutralize partial effect of imatinib on the GIST-T1 cells. In conclusion, these data indicated that CCBE1 may be served as a new predictor of prognosis in post-operative GIST patients and may play an important role in stimulating GIST progression.

Gastrointestinal stromal tumor (GIST), the most major mesenchymal neoplasm of the digestive tract, is characterized by KIT or platelet-derived growth factor receptor alpha (PDGFRA) activating mutations, which approximately account for 80% or 10% of GISTs respectively. GIST is generally believed to derive from interstitial cells of Cajal (the pacemaker cells of the gastrointestinal tract) or related stem cells^1^,^2^, and the most common pathogenic sites are the stomach (60–70%) and small bowel (20–30%)^3^. People over fifty years of age are the highest risk population suffering from GIST^4^,^5^.

The progression of GIST initiates from benign neoplasms and develops to fatal sarcomas, with each step assessed by National Institutes of Health (NIH) grading criteria^1^,^6^,^7^. Traditionally, surgery was the only successful treatment approach for GISTs with a 5 year survival rate of 48–54% 4,8, while patients with irresectable or metastatic disease survived only for a median of 18–24 months after diagnosis with a 5 year survival rate of 5–10%^9^,^10^. Recently, with the development of targeted therapies, imatinib mesylate (also known as Gleevec), a selective inhibitor against mutant forms of type III tyrosine kinases, such as KIT, PDGFRA and ABL, has been used as a standard first-line treatment for irresectable and metastasized GIST patients or adjuvant treatment for advanced GIST patients and has showed dramatically altered in the respect of 5 year survival and recurrence rate^11^,^12^,^13^,^14^. However, 20% of GIST patients with secondary imatinib resistance do not respond to this treatment^15^,^16^,^17^. Thus, to further improve GIST patient survival, it is necessary to uncover the underlying molecular mechanisms of imatinib-induced GIST cell death and secondary resistance.

Extracellular matrix (ECM) proteins, as part of tumor microenvironments, play crucial roles in tumor development and metastases^18^,^19^,^20^,^21^. Given the secretary property, ECM proteins have the potential to be ideal candidates for tumor serum biomarkers and therapeutic targets. CCBE1 is a 44-KD extracellular matrix protein containing an NH2-terminal signaling peptide for extracellular secretion, two repeated collagen domains and two repeated calcium-binding EGF domains. CCBE1 was originally found in a screen for scanning copy number and gene expression on the 18q21-qter chromosomal region in the breast and prostate cancer cell lines^22^. At present, the research of CCBE1 is mainly focused on lymphangiogenesis as a secreted lymphangiogenic factor. It has been reported that CCBE1 is required for lymphangioblast budding and angiogenic sprouting from venous endothelium during embryogenesis in zebrafish^23^. Mutation in CCBE1 would cause Hennekam syndrome, an autosomal recessive disorder, which was characterized by Lymphedema, lymphangiectasias, mental retardation and unusual facial characteristics^24^,^25^,^26^. Recent studies showed that CCBE1 could be transcriptionally regulated by atypical E2f7/8 transcription factor^27^ and positively modulate lymphangiogenesis through promoting the formation of mature VEGF-C from pro-VEGF-C 28,29,30. As well, there are reports showing that loss of CCBE1 impairs erythroblastic island formation and function of fetal liver^31^ and CCBE1 is essential for the migration and proliferation of cardiac precursors cells during early heart development in chick^32^. As for tumor, no research was performed about CCBE1 except for ovarian cancer. In ovarian cancer, CCBE1 is frequently inactivated caused by aberrant promoter hyper-methylation^33^. However, the function of CCBE1 is not completely understood, the clinical significance and effect of the alterations of CCBE1 expression in GIST remain unclear.

In this study, we first explored the expression level of CCBE1 in GIST tissues with different risk degree and its relationship with the clinicopathological characteristics and prognosis. Then, we tested whether the recombinant CCBE1 (rCCBE1) protein can promote angiogenesis of GIST. Lastly, we assessed the effect of imatinib on the viability of GIST-T1 cell in the presence or absence of CCBE1 protein.

## Result

### The expression of CCBE1 is gradually up-regulated in accordance with GIST risk grades

**Figure 1:**
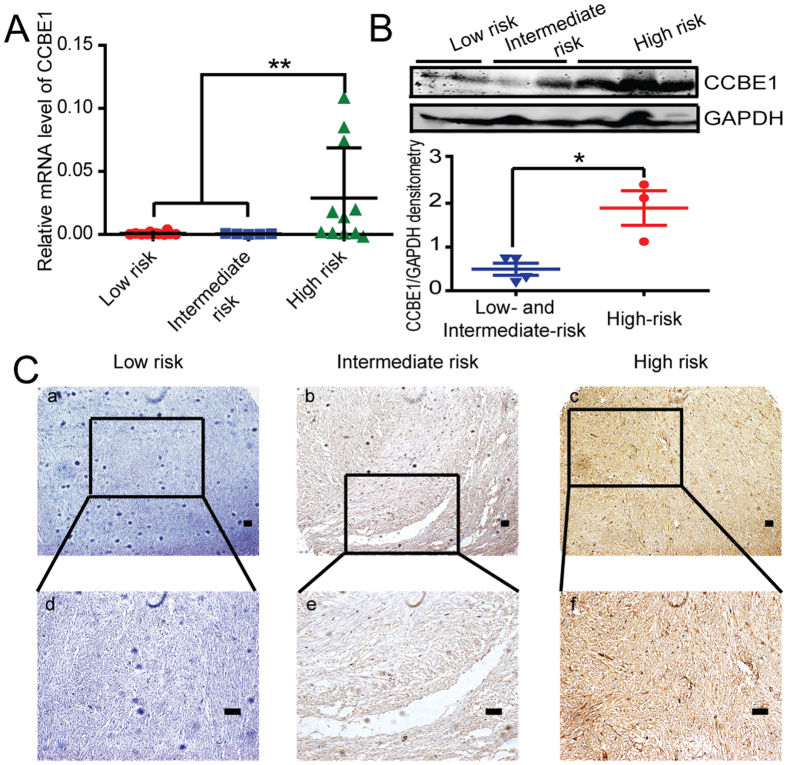
The expression of CCBE1 is gradually up-regulated in accordance with GIST risk grades. (**A**) Relative mRNA expression of CCBE1 in high-risk group was significantly higher than those in the intermediate- and low-risk groups, Values are means ± SEM (**P < 0.01). (**B**) Western blotting analysis showed CCBE1 expression was higher in high-risk samples than the intermediate- and low-risk samples. The densitometric analysis of the results was shown below (*P < 0.05). Glyceraldehyde-3-phosphate dehydrogenase (GAPDH) was included as a loading control. (**C**) Immunohistochemical staining showed CCBE1 expression in high-, intermediate- and low-risk samples. (Original magnification: a–c, 100×; d–f, 200×. Scale bars, 200 μm).

To analyze the expression level of CCBE1 in GIST of different risk grades, we firstly examined the mRNA expression level in human GIST samples by real time-PCR. The results showed that the expression of CCBE1 in GIST tumor tissues of the high risk groups was significantly higher than that of intermediate- and low-risk groups (Fig. 1A). The protein level of CCBE1 was also higher in high risk GIST patients than that in intermediate- and low-risk samples, detected by both western blotting and immunohistochemical staining (Fig. 1B,C).

### Association between CCBE1 protein expression and clinicopathological characteristics of GIST patients

**Figure 2:**
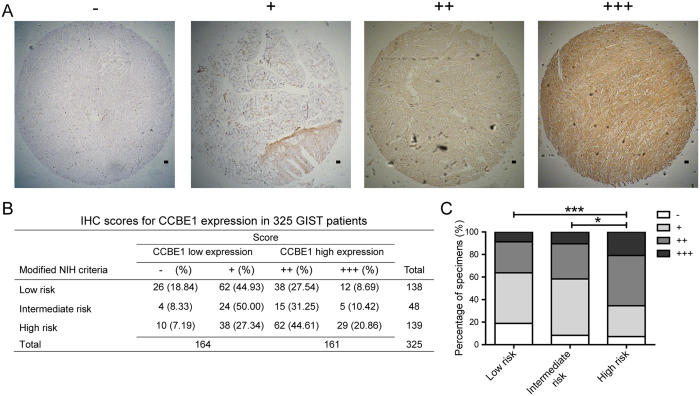
IHC scores for CCBE1 expression in 325 GIST patient tumor tissues. (**A**) Representative immunohistochemical staining for CCBE1 in GIST specimens (Original magnification: 50×. Scale bars, 200 μm). (**B**) Immunohistochemical scoring distribution of CCBE1 in 325 cases. (**C**) Statistical analysis of immunohistochemical scoring distribution in different risk patients (***p < 0.001, *p < 0.05).

To evaluate the clinical significance of CCBE1 expression in GIST, immunohistochemistry (IHC) was performed in a set of GIST tissue microarray including 325 cases. IHC scores of tissue samples according to the intensity and ratio of positive-staining cells were defined as ‘−’, ‘+’, ‘++’ and ‘+++’ (Fig. 2A). Analysis of the staining results showed that 161/325 cases were CCBE1 high expression and 164/325 cases were CCBE1 low expression. In high risk patients, the ratio of patients with higher CCBE1 expression (score as ‘++’ and ‘+++’) was significantly increased compared with that in intermediate- and low-risk patients (63.47% versus 36.13%) (Fig. 2B,C). Then the Chi-square test was used to assess the correlations between CCBE1 expression and clinicopathologic parameters (including age, gender, NIH risk degree, tumor size, tumor site, mitotic figures, tumor bleeding, Ki67 classification, recurrence, and NIH invasion). The results demonstrated that the expression level of CCBE1 in GIST tissues was closely correlated with NIH risk degree (p < 0.001), tumor size (p < 0.001), tumor site (p = 0.012), mitotic figures (p = 0.008), Ki67 classification (p = 0.005), recurrence (p = 0.001) and invasion (p < 0.001). No significant associations were observed between CCBE1 expression and age, gender or tumor bleeding (Table 1).

### Upregulated CCBE1 protein predicts poor prognosis of GIST patients

**Figure 3:**
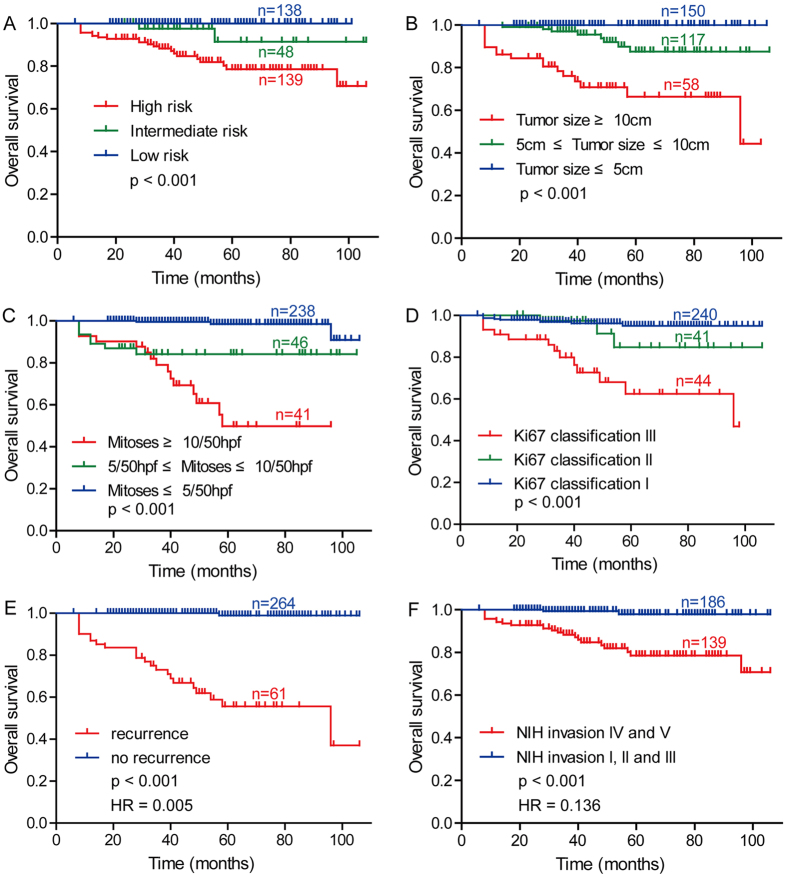
Analyze of the correlation between clinicopathologic parameters and overall survival by Kaplan-Meier method in GIST patients. Kaplan-Meier analysis of overall survival related to (**A**) modified NIH criteria; (**B**) tumor size; (**C**) mitoses; (**D**) Ki67 classification; (**E**) recurrence; (**F**) NIH invasion. HR: Hazard Ratios.

**Figure 4:**
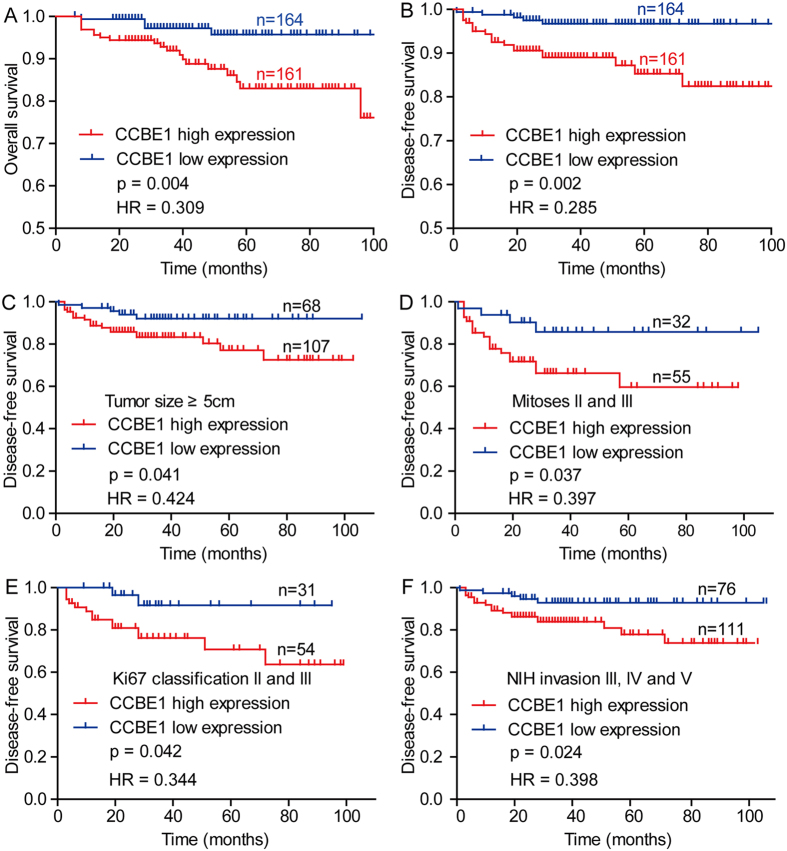
Kaplan-Meier analyzes the association between CCBE1 expression and patient prognosis based on TMA-IHC analysis. (**A**) Kaplan-Meier analysis of overall survival related to the expression of CCBE1 in 325 GIST patients. (**B**) Kaplan-Meier analysis of disease-free survival related to the expression of CCBE1 in 325 GIST patients. (**C**–**F**) Comparisons of disease-free survival between in CCBE1 low expression and CCBE1 high expression in tumor size ≥5 cm cohort, mitoses II and III cohort, Ki67 classification II and III cohort, NIH invasion III, IV and V cohort, respectively.

Before we investigated the relationship between CCBE1 expression and patient prognosis, we firstly analyzed the relationship between clinicopathologic parameters and the overall survival in 325 cases using Kaplan-Meier analysis. The results demonstrated that modified NIH criteria, tumor size, mitoses, Ki67 classification, recurrence, NIH invasion were predictors of overall survival in the GISTs (Fig. 3A–F). Following, we tested the correlation between CCBE1 expression with overall survival (OS) and disease-free survival (DFS) in GISTs, finding that the OS and DFS in the CCBE1 low-expression group were significantly superior to that in CCBE1 high-expression group (Fig. 4A,B). In addition, in the subgroups of patients whose tumor size ≥5 cm, mitoses II and III, Ki67 classification II and III or in NIH invasion III, IV and V, the DFS of CCBE1 low expression group was also superior to that of CCBE1 high expression group (Fig. 4C–F). Univariate Cox proportional hazards regression analysis indicated that CCBE1 expression, modified NIH criteria, tumor size, mitoses, NIH invasion, recurrence and Ki67 classification were hazardous prognostic factors for the overall survival of GIST patients. Meanwhile, multivariate Cox proportional hazards regression analysis identified tumor size, mitoses and recurrence as independent predictors of the OS (Table 2). Taken together, above analysis suggested that CCBE1 was a prognostic factor for the OS of GIST patients, but was not an independent prognostic factor.

### The relationship between CCBE1 expression and the efficacy of Imatinib adjuvant treatment

**Figure 5:**
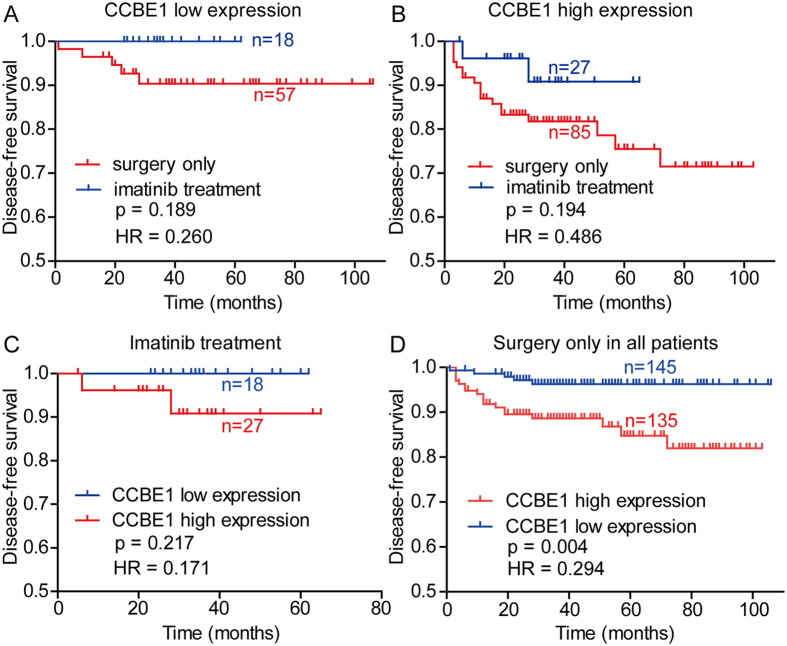
The relationship between CCBE1 expression and the efficacy of Imatinib adjuvant treatment. (**A**) Among CCBE1 low expression intermediate- and high risk GIST patients, there was no significant difference in disease-free survival between the groups with or without imatinib adjuvant treatment. (**B**) Among CCBE1 high expression intermediate- and high risk GIST patients, there was no significant difference in disease-free survival between the groups with or without imatinib adjuvant treatment. (**C**) Among imatinib treatment intermediate- and high risk GIST patients, there was no significant difference in disease-free survival between the CCBE1 high and low expression groups, but a slight decrease of DFS in CCBE1 high expression groups compared with CCBE1 low expression groups. (**D**) In all patients with surgery treatment only, patients had a poorer DFS in CCBE1 high expression groups compared with CCBE1 low expression groups (p = 0.004).

According to the NIH classification guideline, patients with intermediate- or high-risk GIST require adjuvant treatment with imatinib. In this study, we analyzed the correlation between the efficacy of Imatinib adjuvant treatment and the CCBE1 expression level in intermediate- or high-risk GIST patients. The result showed that the DFS rate in the imatinib treatment group were higher than that in the surgery only group, but there was no statistically significance in DFS differences between Imatinib adjuvant treatment samples and surgery only samples in CCBE1 low expression groups (Fig. 5A, p = 0.189) or CCBE1 high expression groups (Fig. 5B, p = 0.194). In patients with imatinib treatment, there was also no significant differences in DFS between CCBE1 high and low expression groups (Fig. 5C, p = 0.217). However, the DFS of CCBE1 low expression group was little longer than that of high expression group. Furthermore, in all GIST patients with surgery treatment only, we found that CCBE1 high expression group has a poorer DFS than CCBE1 low expression group (Fig. 5D, p = 0.004). Therefore, the expression of CCBE1 cannot predict the efficacy of imatinib adjuvant treatment in our current study.

### CCBE1 promotes vessel formation

**Figure 6:**
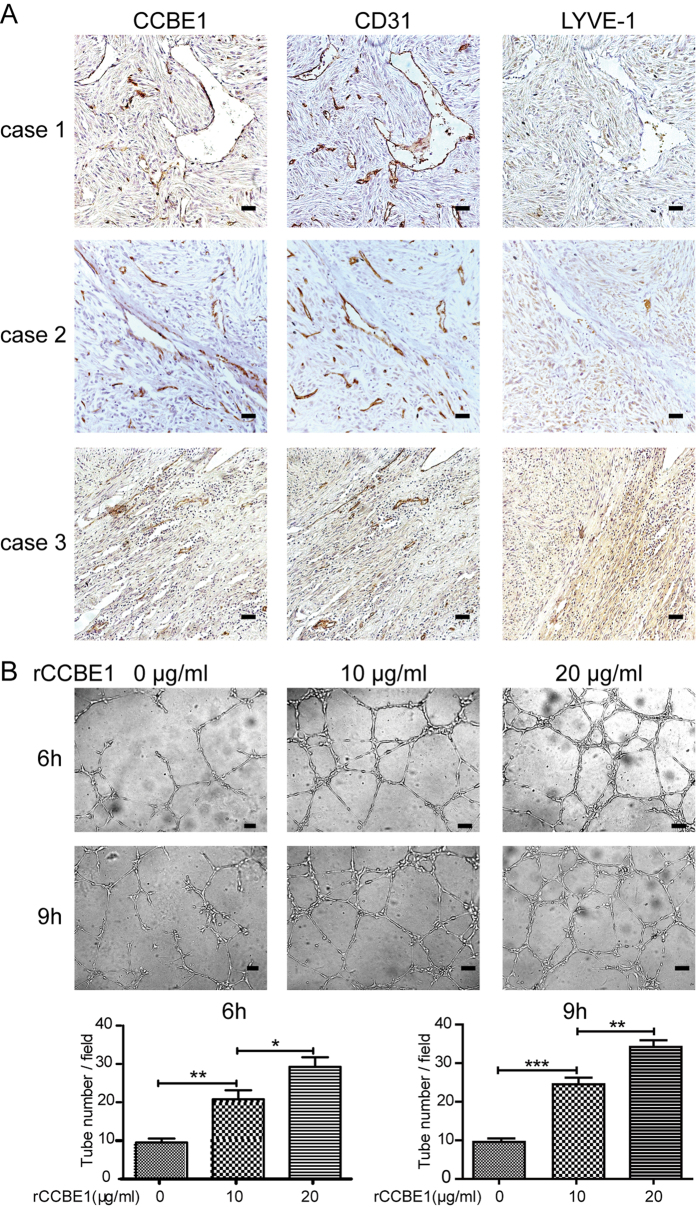
The effect of CCBE1 protein on angiogenesis. (**A**) IHC in consecutive sections of GIST tumor tissues showed that CCBE1 co-localized in the blood vessels wall with vascular marker CD31, nor with lymphatic marker LYVE-1 (Original magnification: 200×; Scale bars, 200 μm). (**B**) Tube formation assay showed that rCCBE1 protein could promote angiogenesis in concentration-dependent manner *in vitro*, tube number per field was measured to reflect the pro-angiogenic effect of rCCBE1 (Original magnification: 100×; Scale bars, 200 μm; ***p < 0.001, **p < 0.01, *p < 0.05).

Previous studies have revealed that CCBE1 modulates lymphangiogenesis and venous sprouting through promoting the formation of mature VEGF-C from pro-VEGF-C 28,29. It has also been shown that the mature VEGF-C can stimulate angiogenesis^30^. In human GISTs, we found that CCBE1 was specifically located in the vessel wall of the tumor tissues (Supplementary Figure 1A). Furthermore, we confirmed that CCBE1 was co-localized with CD31 (a marker of vascular endothelial cells), but not with LYVE-1 (lymphatic vessel endothelial hyaluronan receptor 1, a marker of lymphatic endothelial cells), by IHC in serial sections of GIST tumor tissues (Fig. 6A). To test whether CCBE1 contributed to tumor angiogenesis, the *in vitr*o tube formation assay was performed. We treated the Human Umbilical Vein Endothelial Cells (HUVECs) with recombinant CCBE1 (rCCBE1) protein. The results indicated that CCBE1 promoted tube formation in a dose-dependent manner after treatment for 6 h and 9 h (Fig. 6B).

### Imatinib induces CCBE1 upregulation in GIST-T1 cells

**Figure 7:**
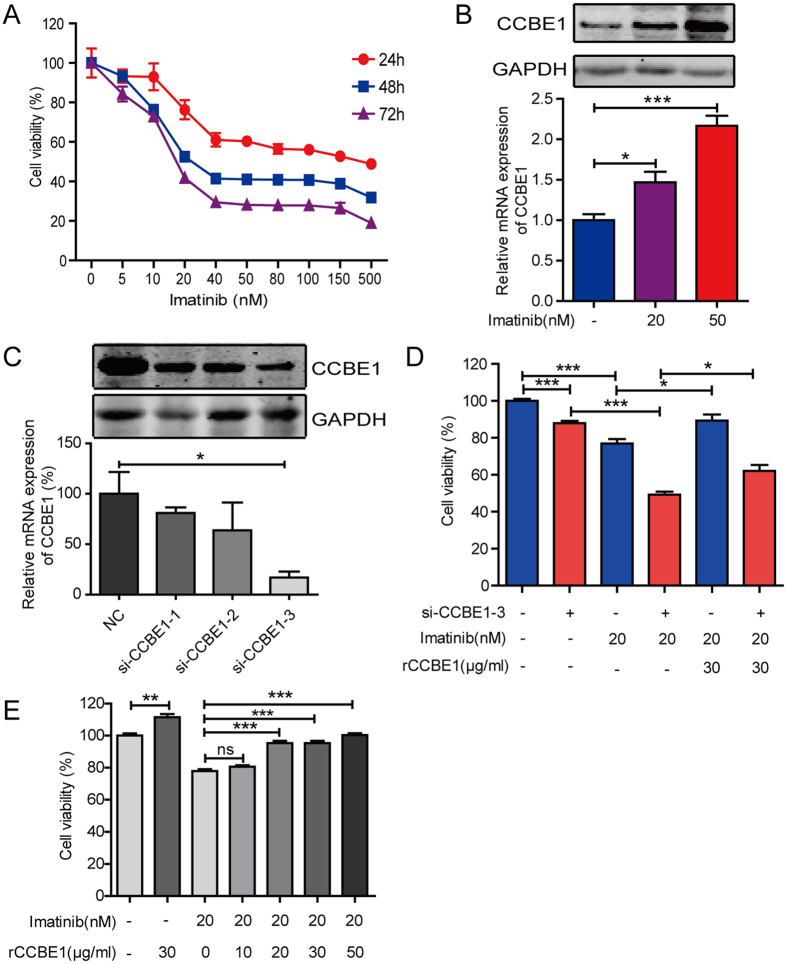
CCBE1 partially reduces the anti-tumor effects of imatinib on GIST-T1 cells. (**A**) GIST-T1 cells were treated with different doses of imatinib for 24, 48, or 72 hours and then cells viability was detected by CCK8 assay. (**B**) After treatment with 20 or 50 nMimatinib for 48 hours, CCBE1 expression was upregulated at both protein (up) and mRNA (below) level. (**C**) Interference efficiency of CCBE1 at protein (up) and mRNA (below) level was measured, respectively. (**D**) Cell viability detected by CCK8 assay after si-CCBE1–3 (20 nM) transfection, imatinib treatment (20 nM) and/or rCCBE1 treatment (30 μg/ml). (**E**) Cell viability detection after rCCBE1 (30 μg/ml) treatment or combined treatment with imatinib (20 nM) and gradient dose rCCBE1 from 0 to 50 μg /ml. ***p < 0.001, **p < 0.01, *p < 0.05.

To investigate the effect of imatinibon CCBE1 expression, we analyzed the dataset (GSE22433) and found that CCBE1 expression was elevated in GIST882 cells when treated with 1 μM imatinib for 8 hours (Supplementary Figure 2). In this study, GIST-T1 cells, one of the imatinib-sensitive GIST cell lines, were used for validating the change of CCBE1 expression after imatinib treatment. We found imatinib treatment resulted in a decrease of viability of GIST-T1 cells and the response appeared to reach a plateau at a concentration of 40 nM (Fig. 7A). Consistent with the data from GSE22433, both mRNA and protein levels of CCBE1 were elevated in GIST-T1 cells when treated with 20 nM and 50 nM imatinib (Fig. 7B).

### CCBE1 partially reduces the anti-tumor effects of imatinib on GIST-T1 cells

To investigate whether CCBE1 could alter GIST-T1 response to imatinib, we first silenced the expression of CCBE1 with RNA interference (Fig. 7C). We used the si-CCBE1-3 for the further experiments. As showed in Fig. 7D, silencing of CCBE1 significantly reduced the cell viability of GIST-T1. Notably, the cytotoxicity of imatinib was amplified by knockdown of CCBE1, and partially inhibited by stimulation with rCCBE1 in a dose-dependent manner (Fig. 7D,E). Taken together, we concluded that CCBE1 could partially counteract the anti-tumor effects of imatinib in GIST-T1 cells.

## Discussion

In this study, we investigated the potential role of CCBE1 as a new predictor of prognosis in post-operative GIST patients and a mediator of drug resistance to imatinib mesylate in GISTs. In the study of CCBE1 expression in 325 patient tissues, we found that the CCBE1 expression was positively related with NIH classification, tumor size and the number of mitotic figures. These indicated that CCBE1 high expression may promote GIST malignant behavior. The overall survival and relapse-free survival analysis revealed that the patients with CCBE1 high expression had poorer overall survival and relapse-free survival than CCBE1 low expression patients. Multivariate Cox proportional hazards regression analysis showed no statistical significance between CCBE1 expression and the overall survival. Thus, we speculated that CCBE1 can be used as a predictor for the overall survival but not as an independent prognostic factor in GIST patients. Of note, in the analysis of the relationship between CCBE1 expression and the efficacy of imatinib adjuvant treatment, we found a relatively good DFS trend in the CCBE1 low expression group with imatinib treatment. As well, we found that CCBE1 could partially reduce the anti-tumor effects of imatinib on GIST-T1 cells *in vitro* assay, which is consistent with the trend. However, no statistically significance was revealed in DFS differences between CCBE1 low expression groups and CCBE1 high expression groups after imatinib treatment. This may be due to limited number of specimens with imatinib treatment. Or the impact would not generate due to the mechanism by which CCBE1 acts in the complex context *in vivo*.

Initially, CCBE1 expression was assessed at mRNA and protein level. The data confirmed that CCBE1 expression was gradually elevated along with the risk degree. Furthermore, CCBE1 protein level as analyzed by IHC showed that the percentage of CCBE1 with high expression in high-risk patients tissues was larger than that in intermediate- or low-risks (65.47% versus 41.67% or 36.23%). Together, these observations indicated that CCBE1 may function as oncogene in GIST, which is inconsistent with the previous reports in the breast cancer^22^ and ovarian cancer^33^. These studies suggest that the regulation and function of CCBE1 maybe vary in different cancer types, similar to Notch receptors, which can function as oncogenes in some contexts and tumor suppressors in others^34^,^35^,^36^. While, in the IHC analysis, the CCBE1 expression was observed not only on the membrane, but also in the cytoplasm. This may be related with its intracellular synthesis before transferring out of the cell in a mature form, like Gal-9, which localized both in the cytoplasm and on the cell surface^37^,^38^,^39^. Or CCBE1 also exerts some functions in the cytoplasm and additional studies need to be performed for verification.

Up to now, the main biological function of CCBE1 was considered as a critical lymphangiogenic factor. Mutations of CCBE1 resulted in failure of lymphatic vessels formation in zebrafish^23^ and cause Hennekamsyndrome in human^24^,^25^. In our study, immunohistochemical staining showed that CCBE1 protein localized specifically to the vessel walls. Moreover, CCBE1 was co-localized with the blood vascular marker CD31 in the blood vessel wall, but not with the lymphatic marker LYVE-1. These data indicate that CCBE1 is expressed in blood vessels in these tumors, but not lymphatic vessels in these tumors. Furthermore, angiogenesis assay showed that rCCBE1 protein enhanced the capacity of tube formation *in vitro*. And we also found that tumors had a higher density of vessels per unit area in high risk patients than in intermediate- or low-risks (supplementary Fig. 1B), which was consistent with the CCBE1 expression levels. However, we didn’t test the angiogenic effect of CCBE1 *in vivo*. In future studies, we will modulate the CCBE1 protein level within GIST xenograft models in order to investigate whether CCBE1 controls angiogenesis in these models *in vivo*.

The expression of CCBE1 was significantly increased upon treatment with 1 μM imatinib in GIST882 cells by analysis of GEO datasets (GSE22433) (Supplementary Figure 2). Inspired by this, we hypothesized that CCBE1 might have some effects on GIST response to imatinib treatment. We first confirmed this in GIST-T1 cell and the similar result was obtained. This may be caused by transcriptionally regulating by atypical E2f7/8 transcription factor induced by imatinib treatment^27^. Then we further manipulated CCBE1 expression levels with RNA interference or treatment with recombinant CCBE1 protein, and found both of modulation exerted significant influence on GIST-T1 response to imatinib. This may be explained by that CCBE1 promote the expression of FOXC2 through VEGFC-VEGFR3-FOXC2 axis^40^ for decreasing drug sensitivity by reducing apoptosis^41^,^42^. However, considering CCBE1 as a secreted protein and the complexity of tumor microenvironment, additional studies need to be performed to figure out the underlying mechanism of how CCBE1 affects GIST response to imatinib. At present, the studies are limited to an *in vitro* cell culture system. The biological functions of CCBE1 in the development and progression of GIST need more *in vivo* studies.

In conclusion, our study demonstrates that CCBE1 expression level may be served as a new predictor of prognosis in post-operative GIST patients. Furthermore, we have shown that rCCBE1 protein can facilitate the potential of angiogenesis and partially reduce the anti-tumor effects of imatinib to GIST cells, suggesting that CCBE1 might be used as a potential clinical therapy target for GIST. However, the molecular mechanisms by which CCBE1 acts in GIST and the possibility of applying this protein as a therapeutic target need to be further elucidated.

## Materials and Methods

### Cell culture and reagents

GIST-T1 cells were obtained from General Surgery of Ren Ji Hospital and cultured in Dulbecco’s modified Eagle medium (DMEM) supplemented with 10% (v/v) fetal bovine serum (FBS) and 1% antibiotics. HUVECs were obtained from the Fifth People’s Hospital of Shanghai and cultured in endothelial cell complete medium containing endothelial cell growth supplement (Allcells; H-004).

### Clinical tissue samples

A total of 325 GIST cases, pathologic diagnosed and treated range from September 2004 to September 2013, were retrospectively identified from the hospitalization archives of Department of General Surgery, Ren Ji Hospital, Shanghai, China. The paraffin-embedded tissue samples of these patients were used for tissue microarray construction and immunohistochemical staining. The study was approved by the Research Ethics Committee of Ren Ji Hospital, School of Medicine, Shanghai Jiao Tong University and carried out in accordance with the ethical standards of the World Medical Association Declaration of Helsinki. All the patients joining this study have signed informed consent. Ethical approval number, 2012031. The inclusion criteria for our study were as follows: 1) an obvious pathologic diagnosis of GIST (CD117 positive in immunohistochemistry staining); 2) primary GIST cases without history of other solid tumors; 3) accepted radical surgery treatment without tumor residual; 4) without any chemotherapy, radiotherapy or other anti-cancer therapies before surgery; 5) availability of complete clinicopathologic and follow-up data; The criterion of imatinib adjuvant therapy is at least twelve months uninterrupted drugs at a dose of 400 mg/day and 45 patients received imatinib adjuvant therapy after surgery before tumor relapse. Patients who received imatinib later for recurrent disease were not included in the imatinib treated group because these patients did not received adjuvant imatinib before the tumor relapse and were regarded as patients who have received surgery only. All the patients involved in our research received physical examination once a month during the first year after surgery and every six months thereafter. High risk GIST patients were accepted computed tomography (CT) or magnetic resonance imaging (MRI) of abdomen and pelvis at three months intervals during the first three years after surgery, and subsequently at six months intervals until five years after surgery. Complete follow-up data for GIST patients in cohort were available.

### Western blotting

Fresh GIST tissues were lysed in tissue protein extraction reagent (Invitrogen). Primary GIST cells were lysed in Western and IP lysis buffer (P0013, Beyotime, Jiangsu, China) supplemented with 1 mM PMSF (Adamas beta, Shanghai, China). Equal amounts of protein were loaded and separated through SDS-PAGE. The proteins were transferred to a membrane, and the blot was blocked with 10% non-fat milk in TBS. The membranes were incubated overnight at 4 °C using the following antibodies: CCBE1 (Sigma Life Science, USA, HPA041374, 1:1000), glyceraldehyde-3-phosphate dehydrogenase (GAPDH) (Proteintech Group, Chicago IL). After incubating with the IRDye 680 anti-mouse (LI-COR, Lincoln, NE) and IRDye 800 anti-rabbit (LI-COR, Lincoln, NE) secondary antibodies for 1 hour at room temperature, the bands were detected by an Odyssey infrared imaging system (LI-COR, Lincoln, NE).

### Immunohistochemical staining

The protocol of this assay was performed as previously reported^6^,^43^. Antibody used as follows: CCBE1 (Rabbit polyclonal antibody, Sigma Life Science, USA, HPA041374, 1:400), CD31(Rabbit polyclonal antibody, Abcam, UK, ab28364, 1:100) and LYVE-1 (Rabbit polyclonal antibody, Abcam, UK, ab14917, 1:100). To quantify the level of CCBE1 protein expression, all the sections were observed and photographed with a microscope (Carl Zeiss) and scored according to the staining intensity: no staining scored 0, weakly staining scored 1, moderately staining scored 2 and strongly staining scored 3 and the ratio of positive-staining cells: 0–5% scored 0; 6–30% scored 1; 31–70% scored 2; more than 70% scored 3. The final score was designated using the staining intensity score × the percent of positive cell score as follows: “−” for a score of 0–1, “+” for a score of 2–3, “++” for a score of 4–6 and “+++” for a score of >6; low expression was defined as a total score <4 and high expression with a total score ≥4. All the CCBE1 expression level was quantified by two independent pathologists.

### Quantitative real-time PCR

For complementary DNA (cDNA) synthesis, total RNA of GIST cells and tissues were extracted using RNAiso Plus (Takara, Dalian, China) following the manufacturer’s protocol and purity and concentration of the isolated RNA were measured on NanoDrop ND-2000 spectrophotometer (Thermo Scientific, USA). Then reverse transcription were performed by PrimeScript RT Reagent kit (Takara, Dalian, China) with 500 ng isolated RNA according to the manufacturer’s instruction in GeneAmp PCR System 9600 (Perkin Elmer, Norwalk, CT) at 37 °C for 15 min and 85 °C for 5 sec. The qPCR was subsequently performed with BestarSybrGreen qPCR Mastermix (DBI Bioscience) according to the manufacturer’s instructions using an ABI7500 instrument (Applied Biosystems). 18 s RNA was used as the reference gene for quantification, and relative standard curve was established for every qPCR assay. The data were analyzed using the 2^−ΔCt^ approach. The Gene-specific qRT-PCR primers as follow: 18S-F:TGCGAGTACTCAACACCAACA, 18S-R:GCATATCTTCGGCCCACA; CCBE1-F: ATGGAGGGCATGCATTTTAG, CCBE1-R: TCAATGAATCCAATGGCAGA.

### CCBE1 recombinant human protein expression, purification and verification

The CDS of CCBE1 were cloned into the episomal expression vector V162 (Supplementary Figure 3A) with pCEP-Pu-Strep II-tag (N-terminal) in frame and the sequence of the BM-40 (SPARC/osteonectin) signal peptide downstream of the CMV promoter. Recombinant human CCBE1 protein was expressed in EBNA-293 cells after transfecting reconstructed plasmid by using X-tremeGENE 9 DNA Transfecting Reagent (Roche, Mannheim, Germany). Forty eight hours after transfection, the EBNA-293 cells were screened with puromycin (Sigma-Aldrich, St. Louis, MO) at a dose of 5 μg/ml in DMEM supplemented with 10% FBS for two weeks, then collected the culture media and Purified by the Strep Tactinsepharose column(IBA, Gottingen, Germany). The purification was performed according to the manufacturer’s protocol, quantified by Nanodrop 2000 spectrophotometer (Thermo Fisher Scientific, Wilmington, DE) and identified by western blotting assay (mentary Figure 3B,C). Cells were exposed to rCCBE1 for 24 hours before detecting by CCK8 assay.

### Vascular tube formation assay *in vitro*

Assay was performed with *in vitro* angiogenesis assay kit (Millipore, ECM625) according to the manufacturer’s protocol. A total of 1 × 10^4^ cells per well were seeded into the 96-well tissue culture plate. Cells in three divided groups were added rCCBE1 protein followed gradient doses of 0, 10 and 20 nM respectively. Inspect tube formation after incubating for 6 h and 9 h at 37 °C, 5% CO_2_ condition.

### RNA interference-based gene knockdown experiment

Cells were transiently transfected with pre-designed small interfering RNA (siRNA) for CCBE1 silencing, the following target siRNA sequences were used: si-CCBE1-1: 5′-CCUGAUCUGUCCCACAUUA-3′;

Si-CCBE1-2: 5′-GACCUGGGCAAGUAUAUCA-3′;

Si-CCBE1-3: 5′-GCCAUGAGAAGUCUGAGAA-3′. A non-targeted (NT) siRNA as a control: 5′-UUGGAGCGUGCGUAAGUAU-3′. The siRNA duplexes were designed and purchased from GenePharma and were transfected into cells at 20 nM per well according to the manufacturer’s instructions using the LipofectamineRNAiMAX Reagent (Invitrogen, Cat. No. 13778-150). After 48 hours, cells were exposed to imatinib and/or CCBE1 for an additional 24 hours before being assayed for changes in CCBE1 expression or in cell viability.

### Cell Viability Assay

GIST-T1 cells were seeded into a 96-well plate (100 μl per well) at 5 × 10^3^ cells per well cultured at 37 °C. At the indicated end point with imatinib or rCCBE1, 10 μl Cell Counting Kit-8 (CCK-8, WST-8, Dojindo, Japan) was added to each well and the absorbance was detected at 450 nm using a microplate reader. The experiment was performed in triplicate and repeated triple.

### Statistical Analysis

Data was presented as the means ± standard error of the mean (SEM). Statistical analyses were operated using SPSS 20.0 software (Chicago, IL, USA). We performed chi-squared tests in cross tables to identify the correlation between CCBE1 expression levels and clinicopathological parameters. Overall survival (OS) and Disease-free survival (DFS) were assessed using Kaplan-Meier method and survival distributions were compared through log-rank test. Student’s t-test was used for comparison between groups. All statistical tests were two-sided. P value less than 0.05 was considered statistically significant.

## Additional Information

**How to cite this article**: Tian, G.-A. *et al*. CCBE1 promotes GIST development through enhancing angiogenesis and mediating resistance to imatinib. *Sci. Rep.* **6**, 31071; doi: 10.1038/srep31071 (2016).

## Supplementary Material

Supplementary Information

## Figures and Tables

**Table 1:** Correlations between CCBE1 expression and clinicopathologic features in patients with gastrointestinal stromal tumor (GIST).

Clinicopathological feature		Total 325	Expression of CCBE1		
			Low (n = 164, 54.3%)	High (n = 161, 45.7%)	P value (χ^2^ test)
Age (years)	≤59	169	87 (51.5)	82 (48.5)	0.702
	>59	156	77 (49.4)	79 (50.6)	
Gender	Male	174	85 (48.9)	89 (51.1)	0.533
	Female	151	79 (52.3)	72 (47.7)	
Modified NIH criteria	Low risk	138	88 (63.8)	50 (36.2)	**<0.001**
	Intermediate risk	48	28 (58.3)	20 (41.7)	
	High risk	139	48 (34.5)	91 (65.5)	
Tumor size	≤2 cm	30	25 (83.3)	5 (16.7)	**<0.001**
	2–5 cm	120	71 (59.2)	49 (40.8)	
	5–10 cm	117	52 (44.4)	65 (55.6)	
	≥10 cm	58	16 (27.6)	42 (72.4)	
Tumor site	stomach	174	94 (54.0)	80 (46.0)	**0.012**
	Small bowel	107	44 (41.1)	63 (58.9)	
	colon	16	13 (81.3)	3 (18.7)	
	others	28	13 (46.4)	15 (53.6)	
Mitoses per 50 HPFs	≤5	238	132 (55.5)	106 (44.5)	**0.008**
	5–10	46	19 (41.3)	27 (58.7)	
	≥10	41	13 (31.7)	28 (68.3)	
Tumor bleeding	yes	53	26 (49.1)	27 (50.9)	0.823
	no	272	138 (50.7)	134 (49.3)	
Ki67 classification	I	240	133 (55.4)	107 (44.6)	**0.005**
	II	41	18 (43.9)	23 (56.1)	
	III	44	13 (29.5)	31 (70.5)	
Recurrence	truncation	264	145 (54.9)	119 (45.1)	**0.001**
	recurrent	61	19 (31.1)	42 (68.9)	
NIH invasion	1	26	21 (80.8)	5 (19.2)	**<0.001**
	2	112	67 (59.8)	45 (40.2)	
	3	48	28 (58.3)	20 (41.7)	
	4	88	29 (33.0)	59 (67.0)	
	5	51	19 (37.3)	32 (62.7)	

Abbreviations: HPF, high power field of the microscope; NIH, National Institutes of Health.

*Median age of total 325 patients was 59 years.

Values in parentheses indicate percentage values. The bold number represents the P–values with significant differences calculated by χ^2^ test.

**Table 2:** Univariate and multivariate analyses of prognostic parameters for survival in patients with gastrointestinal stromal tumor (GIST).

Prognostic parameter	Univariate analysis			Multivariate analysis		
	HR	95% CI	P value	HR	95% CI	P value
Expression of CCBE1	4.196	1.574–11.185	**0.004**	1.816	0.642–5.132	0.260
Age	1.691	0.759–3.766	0.199			
Gender	0.424	0.177–1.016	0.054			
Modified NIH criteria	7.930	2.394–26.268	**0.001**	0.398	0.064–2.475	0.323
Tumor Size	6.902	3.382–14.083	**<0.001**	2.689	1.014–7.129	**0.047**
Tumor location	1.235	0.833–1.829	0.293			
Mitoses per 50 HPFs	5.284	3.195–8.738	**<0.001**	3.060	1.454–6.437	**0.003**
Tumor bleeding	0.399	0.094–1.696	0.213			
NIH invasion	3.701	2.198–6.231	**<0.001**	0.924	0.345–2.479	0.875
Recurrence	238.780	30.701–1857.105	**<0.001**	164.389	13.218–2044.392	**<0.001**
Ki67 classification	3.157	2.033–4.902	**<0.001**	0.915	0.571–1.467	0.712

HR: Hazard ratio; CI: Confidence interval. The bold number represents the P-values with significant differences.
