# Supplementary material for: CCBE1 promotes GIST development through enhancing angiogenesis and mediating resistance to imatinib
Source: Sci Rep. 2016 Aug 10;6:31071. doi: 10.1038/srep31071 (PMC4978997; doi:10.1038/srep31071)
Supplement: Supplementary Information [file srep31071-s1.pdf]

CCBE1 promotes GIST development through enhancing angiogenesis and mediating resistance to imatinib

Guang-Ang Tian[1,3,+], Chun-Chao Zhu[2,+], Xiao-Xin Zhang[1,+],Lei Zhu[1], Xiao-Mei Yang[1], Shu-Heng Jiang[1,3], Rong-Kun Li[1], Lin Tu[2], Yang Wang[1,3], Chun Zhuang[2], Ping He[1], Qing Li, Xiao-Yan Cao[1], Hui Cao[2*], Zhi-Gang Zhang[1*]

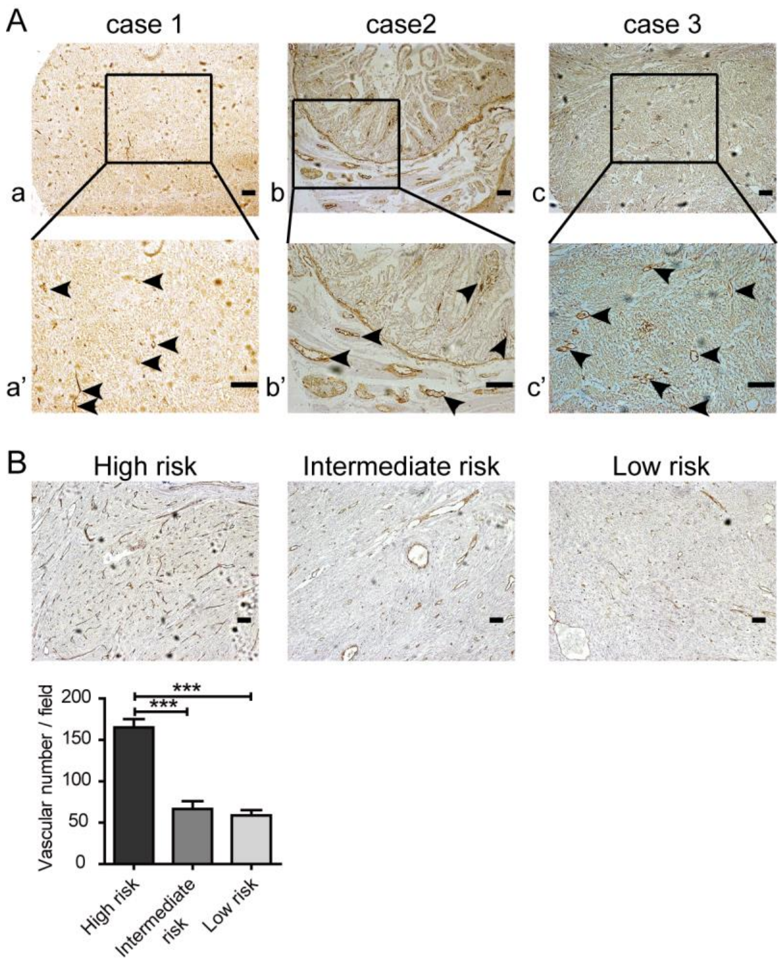

Supplementary Figure 1. The location of CCBE1 in vessel wall of GIST patients tumor tissues. (A) CCBE1 expression specifically located on vessel wall. Thick black arrows in pictures point the vessel wall (Original magnification: 100× and 200×; Scale bars, 200μm). (B) Blood vessels were marked by CD31 antibody in GIST patients tumor tissues with different risk degree. Vascular number was more commonly observed in high risk GIST patients tissues than that in intermediate- and low-risk GIST patients tissues (Original magnification:100×; Scale bars, 200μm).

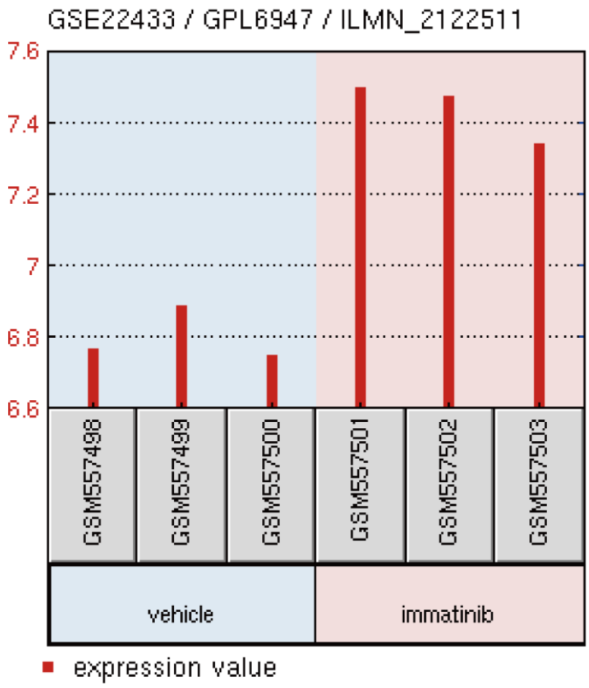

Supplementary Figure 2. CCBE1 change responded to imatinibin GIST882 cell. The expression of CCBE1 was higher increased in GIST882 cells treatment with 1μM imatinib than treatment with0.1% DMSO for 8 hours.

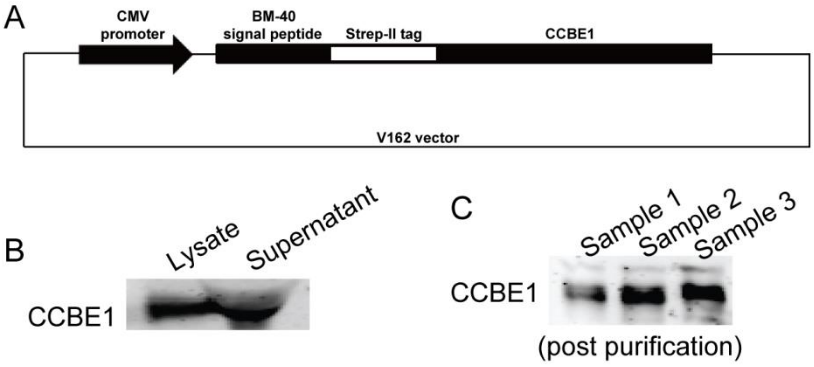

Supplementary Figure 3. Verification of recombinant human CCBE1 protein. (A)The CDS of CCBE1 which removed the nature signal peptide was cloned into the V162 vector. (B) Validation of recombinant CCBE1 protein from the supernatantand cell lysate of HEK-293 cell. (C) Western blotting analysis of the recombinant CCBE1 protein purified from the cell supernatant.

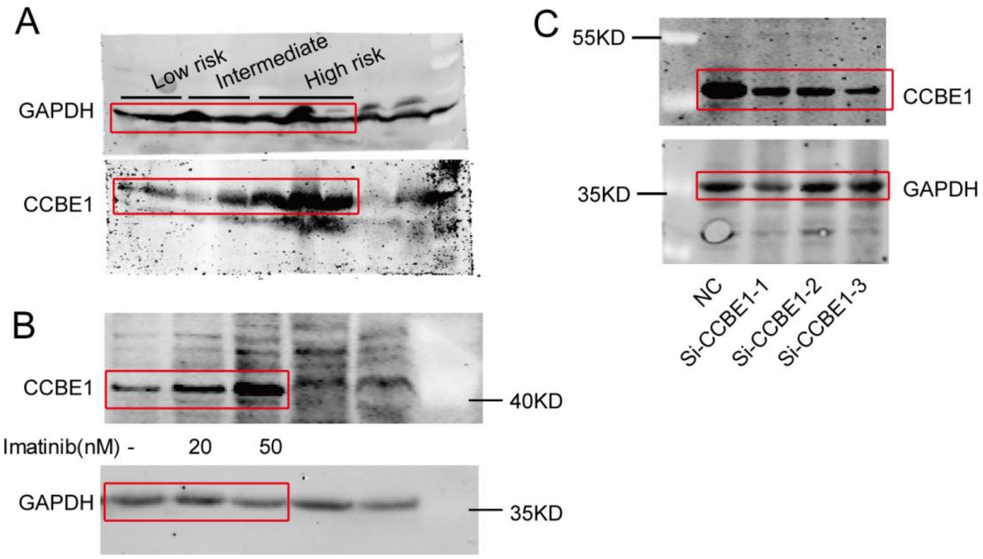

Supplementary Figure 4. Uncropped blots. (A) Blots from Figure 1 panel B. (B) Blots from Figure 7 panel B. (C) Blots from Figure 7 panel C.
